# Empirical Framework for a Relative Sustainability Evaluation of Urbanization on the Water–Energy–Food Nexus Using Simultaneous Equation Analysis

**DOI:** 10.3390/ijerph16060901

**Published:** 2019-03-13

**Authors:** Chihhao Fan, Chun-Yueh Lin, Ming-Che Hu

**Affiliations:** Department of Bioenvironmental Systems Engineering, National Taiwan University, No. 1, Sec. 4, Roosevelt Road, Taipei 10617, Taiwan; chfan@ntu.edu.tw (C.F.); d05622001@ntu.edu.tw (C.-Y.L.)

**Keywords:** WEF nexus, simultaneous equation, urbanization, sustainability

## Abstract

The water–energy–food (WEF) nexus attracts much attention due to the elevated public concern regarding environmental conservation and sustainability. As we head into a new era of civilization, population increase and modernized lifestyles have led to an increasing need for water, energy, and food. However, severe hydrological precipitation significantly impacts agricultural harvest, and such influence becomes more apparent under the influence of climate change. Meanwhile, the major method of electricity generation (i.e., fossil fuel burning) has a negative impact on the environment. These inevitable threats are crucial and have to be dealt with for a society on the road towards sustainability. In the present study, an integrated evaluation of the WEF nexus was conducted for two areas with different levels of urbanization using empirical multiple linear regression in a simultaneous equation model (SEM). By incorporating the collected data into the SEM, the weighting coefficient of each identified variable was obtained, and the nexus implication was assessed in model simulation at different scenarios considering the population growth, agro-technology advancement, energy structure improvement, and available water resources. In the simulated results, three observations were found: (1) the rural area is more sustainable than the urban one; (2) the sustainability for both the investigated areas is significantly subject to their water supply and demand; and (3) food production was found to have a less important effect on the sustainable development of the urban area. This study identified the key factors in the WEF nexus exploration, which are economically and environmentally important for resource allocation. An empirical model was developed to correlate sustainable achievement with WEF management, as well as strategic policies that should be implemented under the pressure of urbanization.

## 1. Introduction

Food, water, and energy are indispensable resources for human survival, and they are the key factors maintaining the balance between human activities and the environment. The Stockholm Environment Institute (SEI) firstly proposed that the relationship between water, energy, and food (WEF) is deeply interrelated. When one of these resources is affected by climatic conditions, human activities, or policy implementation, the WEF nexus is disturbed and becomes even more complicated [1]. The International Renewable Energy Agency (IRENA) [2] indicates that energy consumption will double in 2050, and the need for food and water will increase by 50%. Due to climate change, high-intensity rainfall events and long-lasting droughts cause concerning issues, such as water scarcity, agricultural production loss, and extra expenditure for damage recovery. The available resources can barely meet the future demand if the population keeps growing and resources are expended in an inefficient way [3].

WEF is a must for human society development, and the quantitative correlations among these resources have not been investigated to a satisfactory extent [4]. Although urban areas contribute a significant amount of the gross domestic product (GDP) of a nation, agricultural activities in rural areas consume almost 70% of the available water resources and 30% of the electricity. To meet the WEF demands in an urban area, water for agricultural uses is oftentimes transferred to meet industrial and domestic needs. Resource consumption may increase stress on resource allocation, and such a phenomenon occurs frequently in urban areas. Under the influence of climate change, extreme weather hazards limit the availability of WEF resources. For example, drought events can cause shortages in the water supply and food production, as well as energy generation, leading to an economic loss of agricultural productivity [5,6,7].

Therefore, many efforts have been devoted to the exploration of the WEF nexus and have aimed to balance WEF resources to maintain ecosystem services [8], to make the public understand the policy of resource management, and to propose a reliable WEF nexus model using the concepts of material flow, environmental and ecological conservation, social-economic development, and human behavior [9]. Meeting the public demands is no longer the only goal and using these resources more efficiently has to be considered. Through the investigation of the WEF nexus, their dynamic relationship can be explored and used to deal with the dilemma when making a policy at the cost of an acceptable compromise [10], which can benefit the human welfare, as well as the environmental integrity [11].

As international society advances towards a sustainable era, urbanization and resource concentration are also becoming inevitable trends, which dominate resource allocation and increase waste generation. However, global communities have varying definitions of urbanization and adopt different approaches for further exploration. Some of them consider land use for urban expansion and industrial re-structuring and analyze its impact on food production and industrialization [12]; others explore the effect of population increase on urban development with respect to resource allocation and circulation [13]. As the level of modernization diversifies in different communities, the consumption of WEF resources for a given community may exhibit a unique pattern [14]. Resource consumption may vary markedly to meet diverse needs in urban areas and to maintain activities in rural areas. Urbanization can facilitate demographic and societal advancements, which increase social welfare. In the process of urbanization, water and energy consumption increases, which also increases the level of pollution along with the competition for resource utilization; however, unbalanced resource management limits the efficient utilization of resources [15,16,17,18].

Generally, rural areas support agricultural growing activities, which require a substantial amount of water. Despite the fact that agro-growing technology has advanced significantly, water and energy consumption still exhibits an increasing trend. A large population in an urban area consumes water and energy significantly but with a higher efficiency of resource utilization. Meanwhile, urbanization has become an unstoppable trend in developing countries. Hence, finding the solution to maintain the balance of various resource utilization is considered to be a priority issue to be solved [19,20,21].

The increasing population facilitates economic development, because with such trends, more working forces are in the market [3] and more food, water, and energy are consumed [4]. Unfortunately, excess in resource utilization imposes a huge burden on ecological conservation and increases the risk of environmental degradation [22]. Thus, waste treatment and reduction, for example, were considered to be an unavoidable obligation to preserve environmental quality [9]. In the exploration of the WEF nexus, sustainability should include not only reducing energy consumption and waste production, but also providing renewable, clean, and efficient resources [23]. In 2050, the global population is expected to be around 9.8 billion, which will lead to increased WEF consumption. Even though the population continue to grow, the extra labor does not necessarily tend to participate in food-producing activities.

The United Nations Environment Programme (UNEP) has announced the Green Economy Initiative based on the concept of sustainable development, which reconsiders resource distribution from supply management to ever-changing consumption behaviors. Sustainability has become the most popular concept almost in every human-related activity. The United Nations Commission on Environment and Development (UNCED) defined sustainable development as fulfilling current needs without impairing the needs of future generations, which provides applicable guidelines for actions; however, a similarly practical definition for a sustainable WEF nexus has remained undetermined.

The United Nations (UN) proposed 17 goals for achieving sustainability, which include zero hunger, clean water and sanitation, affordable clean energy, and many others. In the rural areas of many undeveloped countries, people do not have sufficient electricity nor enough food to meet daily survival demands. To alleviate this situation, agricultural productivity must be increased to meet the demands of the increasing population. To encourage cultivating and farming activities, a farmer’s earnings must be secured at a tempting level to maintain agricultural productivity. Although some countries produce enough food for their own people, there are still many countries that can barely do so. To achieve sustainability, marketing strategies for a reliable, affordable, and profitable food supply system should be constructed. Due to the shortage of the food supply and water resources worldwide, one out of four people will suffer from freshwater shortage in the near future, and the freshwater supply will be the subject of high competition in the agricultural, industrial, and domestic sectors. Approximately 70% of water extracted from natural waterways is used for irrigation. However, more than 80% of agricultural wastewater is discharged into receiving water bodies without proper treatment. Energy has always been the key issue affecting sustainable development. All the processes that produce food and water require energy input despite the fact that power generation can cause serious environmental problems that threaten human safety and health. A sustainable energy supply not only generates enough electricity, but also provides clean energy that inflicts the least harm to the environment.

The core values of the sustainable development goals (SDGs) are to end hunger, achieve food security and improved nutrition, and promote sustainable agriculture, and these goals seem to be the fundamental requirements for a sustainable society. Guaranteeing a sufficient food supply and increasing agricultural productivity and profitable incomes of agro-food producers are possible incentives to encourage agriculture development. Furthermore, enhanced diversity in an ecosystem can strengthen agricultural productivity by increasing its resilience to climatic extremes. Apparently, the WEF nexus is complicated and interrelated, which deserves further exploration to address the issue of WEF resources allocation.

## 2. Nexus Approach and Related Studies

To address WEF issues, many studies have been dedicated to the investigation of the WEF nexus and its impact on society’s development. However, the studies combining these three resources to evaluate society’s advancement toward sustainability remain limited. Most of the WEF nexus research has modeled physical WEF systems and analyzed governances and management systems, which provide a comprehensive picture of the interrelationship of WEF. Unfortunately, the obtained solutions and suggestions have seldom been implemented in practice. The inconsistency in policies and sectoral coordination leads to insufficient resource development and management [24].

Vanham (2016) investigated water management in agricultural, industrial, and domestic uses. To grow agricultural products, 70% of the available water and 30% of the global energy were consumed, and the food-producing technology has been improved to increase productivity. However, the low cost of the water supply and energy held back the investment in developing new technologies, which also caused excessive waste in resource expenditure [5,25].

Zhang et al. (2018) extracted the main focuses in the field of the water–energy–food nexus by reviewing articles in the literature. The selected papers were classified according to their definitions of nexus, research questions, scales, and adopted methods. Challenges for future research were identified by summarizing the limitations of the selected papers [26]. Zhang et al. (2019) conducted a study that provides a comprehensive literature review to debate the current concepts and methods of the WEF nexus at different scales, aiming to develop a conceptual knowledge base framework for scientific analysis and policymaking associated with the urban WEF nexus. Although the concept of nexus thinking has been widely accepted, a consistent and explicit cognition of the WEF nexus is still lacking, and a sophisticated methodological modeling framework is required at various scales [27]. Li et al. (2019) indicated that factors in the WEF nexus vary by time, level, and location, but the hierarchy between factors has been largely ignored. Taking advantage of the interpretive structural modeling (ISM) method, identification and analysis of the interwoven factors in an urban WEF nexus in Beijing were conducted. As a result, 87 representative factors were identified, with a hierarchy structure established by ISM. Based on the relative importance of the given factors, the factor hierarchy structure showed that the energy system in the core nexus is the essential system and is critical to promoting the WEF nexus in Beijing; factors from peripheral nexuses—such as population and vehicle volume—also have a significant influence on nexus governance [28].

Apparently, the WEF nexus is an issue that attracts much attention, and the simultaneous equation has been employed to investigate the nexus relationship among variables. In the study by Tiba and Frikha (2018), the three-way causality between income, trade openness, and energy consumption was examined through the use of simultaneous-equation panel data models (SEPDM) for 24 middle- and high-income countries for the time span of 1990–2011. The empirical results for the high-income countries showed the presence of feedback causality between energy consumption and income and between trade openness and income. These empirical insights are of particular interest to policymakers as they help build sound economic policies to sustain economic development [29]. Lu et al. (2017) used sulfur dioxide emissions, wastewater emissions, and soot and dust emissions as indicators of environmental quality to investigate the comprehensive dynamic relationship between environmental quality, economic development, and public health in China. To control for potential endogeneity, a carefully designed simultaneous equation model (SEM) that is composed of three equations that describe the relationships among economic development, environmental quality, and public health was utilized. Using panel data from 30 Chinese provinces for the period from 2002 to 2014, the model verified the negative effect of environmental pollution on public health [30]. Another example of a simultaneous equation study is that conducted by Hsiao and Zhou (2015). The identification and estimation of dynamic simultaneous-equations panel models were conducted. The presence of time-persistent individual-specific effects does not lead to changes in the identification conditions of traditional Cowles Commission dynamic simultaneous-equations models [31]. Obviously, simultaneous equation can be applied to investigating the nexus relationship among variables, especially when there is a complicated intercorrelation among the parameters.

In the process of solving an equation matrix, many different mathematical algorithms can be employed. In the present study, empirical multiple linear regression was used, and this technique is popularly used in defining the correlation among investigated variables empirically. Therefore, the present study aimed to develop a sustainable index, which shows the WEF nexus quantitatively, and to propose applicable suggestions for future policymaking. An integrated evaluation was conducted for two areas with different levels of urbanization using empirical multiple linear regression in a simultaneous-equations model. Sustainability achievements under the implications of the WEF nexus for several given scenarios were evaluated comparatively and empirically.

## 3. Methodology

### 3.1. Background of Studied Areas

Taiwan is a small island (36,000 km^2^ in area) situated in southeast Asia, with a population of around 23 million and convenient transportation. There are 6 major municipalities, and more than 70% of the total population lives within urban areas. With high population density and limited land resources, Taiwan is vulnerable and sensitive to a variety of disasters; storms, earthquakes, floods, and droughts may pose significant threats to the stable supply of WEF resources [22]. In Taiwan, the mainly consumed crops, such as corn, wheat, and soybean, are imported from other countries. Meanwhile, climate change alters precipitation patterns by increasing the time interval between rainfall events, resulting in the occurrence of drought events frequently [32]. In recent years, limited water supplementation caused by droughts has had a negative impact on agricultural, industrial, and domestic sectors [33]. The power supply in Taiwan is mainly supplied by fuel-based thermal generators at the proportion of 70%, and the other 30% is generated by nuclear reactions, hydropower, and wind and solar power. Obviously, Taiwan’s power generation does not result from environmentally friendly methods. To meet the increasing demands of electricity consumption, a greener and more sustainable method should be incorporated to fulfill the current consumption and to ensure future needs.

Along with the advancement of society, urbanized areas attract most of the labor force due to the increasing opportunities for career development. As a consequence, the farming areas in Taiwan are facing the pressure of transformation into urbanized environments, which may degrade the environmental quality, bring negative impacts to water and food resources, and cause land use consolidation. As a rugged island located in a subtropical region, fragmented farmland and tropical storms limit agriculture development in Taiwan, resulting in small-scale farming with high cost and less productivity. Although innovative agricultural technology improves productivity and quality of agriculture produce, the overall agro-farming industry in Taiwan is still considered to be less competitive compared with those in the countries with large land resources.

In the present study, two areas (i.e., Taoyuan and Yunlin) with different levels of urbanization were selected to investigate the WEF nexus due to resource consumption. Taoyuan used to be one of the major providers of agricultural produce in Taiwan. Over the past few decades, the industry structure changed, because many engineering projects were implemented. Taoyuan became a city full of industrial and commercial activities. By contrast, Yunlin, currently one of the major agricultural producing areas, was chosen as an example of a rural area. Because the weather, the populations and human activities are different in these two areas, their concerns for pursuing sustainable development were expected to be dissimilar.

**Figure 1:**
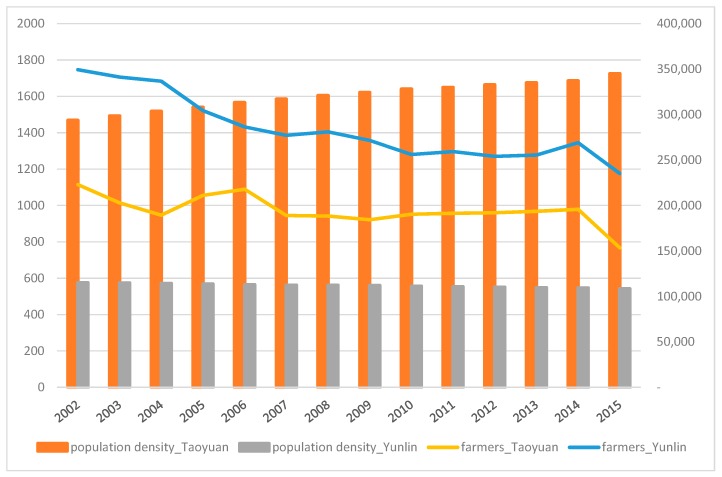
Population density (person per km^2^, left scale) and number of farmers (person, right scale) in Taoyuan and Yunlin, 2002–2014.

**Figure 2:**
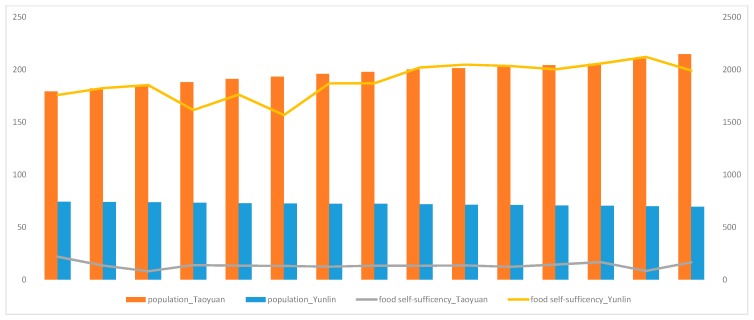
Food self-sufficiency (%, left scale) and population (thousand people, right scale) in Taoyuan and Yunlin, 2002–2014.

The population density of and number of farmers in the two investigated areas are shown in Figure 1. The population density in Taoyuan is more than twice as much as that in Yunlin, implying a higher intensity of land use. The number of people participating in agriculture cultivation decreased, and such change is more apparent in the urban area. As shown in Figure 2, Yunlin, which has a smaller population, can produce more food to support local needs; on the contrary, there are nearly 2 million people in the urban area of Taoyuan in which only 20% of the required food is produced locally.

### 3.2. Parameter Selection

As the purpose of this study is to explore the nexus of water, energy, and food under the stress of urbanization, the sustainable water, energy, and food indices were used as the target functions. By inspecting the available data collected by different governing agencies and administrative authorities, the agro-cultivated production, water resource consumption, and electricity consumption were used to represent the sustainable use of water, energy, and food. One thing that should be noted is that more cultivated production would be considered “more sustainable” and an increase in water and energy demands may result in a “less sustainable” situation. All the data were used as they were collected without further transformation to maintain their genuine characteristics.

#### 3.2.1. Water

Regarding sustainable water resource management, the goal is to provide affordable water for all people. Even though water resources are transferred and stored, water quality becomes another issue under the influence of urbanization. Hazardous materials and untreated wastewater must be treated properly to increase the availability of usable water. The installation of water purification systems can improve contaminant removal and water quality. After meeting the requirement of certain water quality criteria, treated water can be regarded as usable water. The stable water supply, high water-use efficiency, and sufficient water storage systems are fundamental to ensuring sustainable water utilization and should apply applicable innovative technologies. All human activities and industrial manufacturing processes require consuming water. In Taiwan, most of the urban areas have well-planned infrastructures of water supply systems, and many rural areas require the installation of water purification and distribution systems. In sustainable water analysis, access to tap water, water quality, and wastewater treatment were selected as the key parameters. Meeting the public need in accessing tap water is fundamental to ensuring the water resource sustainability. This study employed the river pollution index (RPI) as the indication of water quality. The RPI system is currently employed by the Taiwan Environmental Protection Administration for water quality management, and concentrations of suspended solids, dissolved oxygen, biochemical oxygen demand, and ammonia nitrogen are used to calculate the RPI to represent the degree of pollution of a given water body.

**Figure 3:**
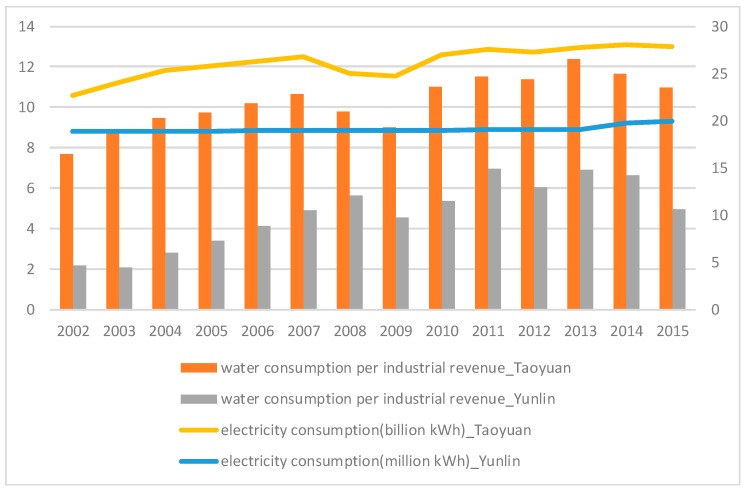
Water consumption per industrial revenue (million m^3^, left scale) and electricity consumption (billion in Taoyuan, million in Yunlin, right scale) in Taoyuan and Yunlin, 2002–2015.

Resource consumption increases as the population grows and the industrial structure advances. In Figure 3, water consumption per industrial revenue of the two selected areas is shown from 2002 to 2014, implying that the increase in water utilization efficiency may be due to the application of innovative technologies.

#### 3.2.2. Energy

Energy is indispensable to modern society as the modern lifestyle increases energy consumption. The goal of sustainable energy development is to ensure energy generation through a stable process with fewer harmful effects to the environment. Despite the technology advancement over the past few decades, safe, clean, and sufficient electricity generation remains to be the ultimate goal of environmental sustainability. Usually, the energy policy is determined by considering domestic natural resources and the balance between the supply and demand of energy. In order to support economic development and to assure the wellbeing of the people, an affordable and reliable power supply is definitely among the top priorities in strategic energy planning. Power supply by traditional thermal generation is the major way to meet the domestic electricity demands. Realizing the inevitable increase of pollution associated with the thermal power generation, green energy, such as solar power and hydropower, has been introduced as possible alternatives. However, many technologies for green energy generation require further exploration and experimentation before they can be implemented as a replacement power supply. Additionally, energy is considered to be a required resource of daily life, and energy prices reflect the structure of the energy-generating process. In this study, the sustainable energy model considered the share of renewable energy and energy price. Similarly, the price of energy has a significant influence on the energy generation. The high energy consumption by the industry will lead to the pursuance of a lower energy price to meet the public need of affordable and sufficient energy.

#### 3.2.3. Food

Different countries consume varied food according to their culture and lifestyles. To assure the food supply, the concept of self-sufficiency is important, in that local production and consumption should be considered simultaneously, by which the expense of food transportation can be reduced. In reports proposed by the UN, sufficient food is a basic requirement, and food price is another influencing factor that has to be considered. The quantity of food supply seems meaningless if its price is not affordable to the public. The proposed model uses agricultural productions, farmer salary, and food prices as the key factors affecting the sustainable food index.

### 3.3. Model Development

**Figure 4:**
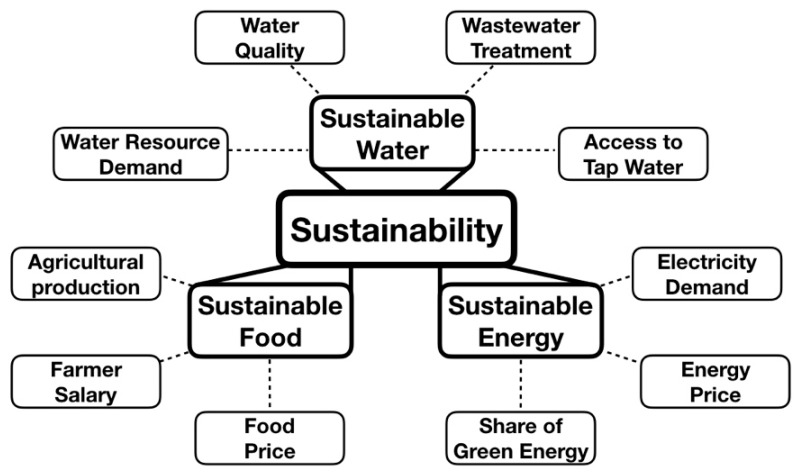
Sustainable water–energy–food (WEF) nexus.

The WEF nexus is a conceptual matrix, with complicated intercorrelations between water, energy, and food, which provides an overall description by examining the projects, policies, and relationships among these resources [34,35,36]. For the complex WEF system, indexing model technique can quantitatively describe a system and optimize its management. Indicators involved in a WEF nexus help to understand how the consumption of WEF resources can affect the production systems [37,38,39]. A well-design index can systematically identify the strengths and weaknesses of policies and help policymakers to justify and improve decisions. Hence, a sustainable WEF index can reduce the excessive abuse of resources and monitor whether the system is sustainable and sufficient enough [40,41,42]. This study employed cross-region data collected over more than a decade to formulate the sustainable WEF model (Figure 4).

Three endogenous variables (sustainable water, SW; sustainable energy, SE; sustainable food, SF) and seven exogenous variables were incorporated in the establishment of the SEM structure, and each equation involves at least one endogenous and two exogenous parameters. In the regression calculation, the data of the two investigated regions (i.e., representations of urban and rural areas) between 2002 and 2015 were collected from the statistical offices of the governing authorities. The descriptive statistical results for the selected rural (Yunlin) and urban (Taoyuan) areas are presented in Table 1 and Table 2, respectively. This study constructed an empirical equation system of sustainable water, energy, and food regression models, using a simultaneous equation system to investigate the interactions between WEF (Equations (1)–(3)). The three major variables were chosen according to the United Nations Sustainable Development Goals (i.e., zero hunger, clean water and sanitation, and affordable and clean energy). To be more specific, food production, water consumption, and energy consumption were chosen as the respective sustainable food, water, and energy variables. For each variable, all the available data in the related category were collected and used for subsequent regression calculation. In this study, seven parameters were considered to be related to the WEF nexus. Although a few other parameters may be available, the data with different collection time periods make it inappropriate to include these parameters into the model calculation.

The ultimate goal of the present study is to form an evaluation framework that is scalable and transferrable. Therefore, a simultaneous equation system with multiple regression analysis was employed in the model derivation. Variables with higher correlation coefficients were included in the derived models. In the practice of multiple linear regression, many examples can be found that the data was utilized in its original form and that the target function was correlated with independent variables empirically, and a few examples can also be found in the statistics textbooks [43]. In these illustrated examples, all the data were regressed numerically to obtain the correlation among them empirically. In this study, a similar calculation concept was employed in the process of model development.

(1) $$\mathrm{SW}=\beta_{0}+\beta_{1}\mathrm{SE}+\beta_{2}\mathrm{AT}+\beta_{3}\mathrm{WQ}+\beta_{4}\mathrm{WT}+\varepsilon_{2}$$

(2) $$\mathrm{SE}=\gamma_{0}+\gamma_{1}\mathrm{SW}+\gamma_{2}\mathrm{RE}+\gamma_{3}\mathrm{EP}+\varepsilon_{3}$$

(3) $$\mathrm{SF}=\alpha_{0}+\alpha_{1}\mathrm{SW}+\alpha_{2}\mathrm{SE}+\alpha_{3}\mathrm{FS}+\alpha_{4}\mathrm{FP}+\alpha_{5}\mathrm{WQ}+\varepsilon_{1}$$

where SW, SE, and SF are sustainable water, sustainable energy, and sustainable food indices, respectively; AT is the service ratio of tap water (defined as the total number of residences with tap water service divided by the total number of residences); WQ is water quality; WT is the ratio of wastewater being treated (defined as the amount of wastewater being treated before discharge divided by the amount of wastewater generated); RE is the share of renewable energy in total power supply; EP is the price of energy; FS is the average farmer salary; FP is food price; α_0_, β_0_, γ_0_ are the regression residuals; $\alpha_{n},{\;\beta }_{n},{\;\gamma }_{n}$ are weighting coefficient; and $\varepsilon_{1}$, $\varepsilon_{2},{\;\varepsilon }_{3}$ are error terms. In this model, SF, SW, and SE are endogenous variables, and the other variables are assumed to be exogenous.

This study proposed a feasible framework of comparative assessment of WEF sustainability. First, 10n related parameters were collected for model development. One WEF variable was regressed with the other nine parameters, and the parameters with insignificant correlation coefficients were eliminated from the model. The regression was performed using the often-used computer program of Statistical Analysis System (SAS) originally developed by North Carolina State University. The regression procedure (REG) was used to estimate the parameters by ordinary or weighted least squares and assume homoscedastic, uncorrelated model errors with zero mean.

Table 1 and Table 2 report the descriptive statistics of the variables used in this study. In the model, Equation (1) delineates the sustainable food index as a function of sustainable water and energy indices, average farmer salary, food price, and water quality. This equation also implicitly implies the agricultural farming requiring the consumption of water and energy. In Equation (2), sustainable water index is determined by combining the effects of sustainable energy, access to tap water, water quality, and the ratio of wastewater treated. In Equation (3), sustainable energy index depends on the sustainable water index, the share of green energy, and energy prices. Because most of the energy-generating systems require cooling processes, water resources are another key factor.

### 3.4. Sustainable WEF Index

To explore the sustainability of the investigated areas, the SF, SW, and SE were incorporated into the sustainable WEF index (SI) as shown in Equation (4). The WEF nexus is about cross-sectoral development and the management of resources, and all the resources should be viewed in equal terms. There is no resource that should be considered to be more important than the others, as the WEF nexus is about interlinkages, interdependences, and interconnectedness of all the sectors. Under such a circumstance, the equation employed the equal weights for water, energy, and food. In the SI calculation, the issue of human sustainable development is the major concern, and the population density is regarded as the intensity of consuming resources. In the urban area, active human activities are consuming remarkable WEF resources, while the rural areas possess more natural landscapes with less resource consumption [44,45].

(4) $$\mathrm{SI}=\ln{\left(\mathrm{PD}\right)}^{\mathrm{SW}+\mathrm{SE}-\mathrm{SF}}$$

where SI is the sustainable WEF index, PD is the density of the population, SF is sustainable food, SW is sustainable water, and SE is sustainable energy. The integrated sustainable WEF index (SI) was proposed by including the logarithmic of population density as the weighting factor. In the two investigated areas, all the resources are consumed to support the survival of the humans, and population density becomes a factor. In a densely populated area, more resources should be consumed, and more waste should be generated as well. However, the amount of consumed resources may not be exactly proportional to the population density. In this model, we proposed that a logarithmic form of population density should be included as the weighting factor in the integrated index to account for the intensity of resource consumption. Once the SI model is established, the SI values for different situations and scenarios can be calculated by substituting the respective data into the developed model. For all the data used for calculation, please refer to the database maintained by the related agencies in Taiwan [46,47,48,49,50]. As mentioned previously, the SW and SE are the water and energy demands of the investigated areas. The increases in SW and SE imply a less sustainable condition and lead to the increase of SI. In this regard, a higher value in SI represents a less sustainable condition.

After the SI model establishment, several scenarios with different policy and project implementations were simulated. The simulation scenarios were chosen based on the standards of the UN for achieving sustainability. The sustainability is considered to be achieved if the following criteria are met: (1) doubling the agricultural production and (2) farmer salary, (3) maintaining stable food prices, (4) increasing the ratio of tap water access more than 90%, (5) improving the water quality to meet the requirements of the clean water category, (6) increasing the ratio of the appropriate wastewater treatment more than 90%, (7) maintaining stable energy price, and (8) increasing the power supply of renewable energy by more than 50%. Three scenarios of enforcing current practice, sustainable practice (the UN standards), and unsustainable practice (opposite implementation of the UN standards) were simulated using the data collected in 2016 as the basis. For the variation of the population, the estimation of the governing agency for a population density decrease of 20% in 2050 for the two areas was adopted.

## 4. Results and Regression Analysis

### 4.1. The Construction of the Simultaneous Equations Model

The WEF regression models are used to describe the sustainability of resource utilization of rural and urban areas, because the environmental and social-economic conditions are different in these areas. Multiple linear regression was used to estimate the weighting coefficient of each considered variable. Table 3 shows the regression coefficients of the selected variables in SF, SW, and SE regression models for the Taoyuan and Yunlin areas, respectively. Equations (5)–(7) are the models representing WEF sustainability in Taoyuan, which is a modernized city with high consumption of resources. Equations (8)–(10) show the correlations between resource sustainability and selected parameters in Yunlin, a rural area with massive agricultural activities.

(5) $${\mathrm{SW}}_{T}=43.65-0.28{\mathrm{SE}}_{T}+0.66{\mathrm{AT}}_{T}-0.27{\mathrm{WQ}}_{T}-0.54{\mathrm{WT}}_{T}$$

(6) $${\mathrm{SE}}_{T}=20.95-0.13{\mathrm{SW}}_{T}+0.69{\mathrm{RE}}_{T}+0.1{\mathrm{EP}}_{T}$$

(7) $${\mathrm{SF}}_{T}=22.43-0.25{\mathrm{SW}}_{T}-0.73{\mathrm{SE}}_{T}+0.21{\mathrm{FS}}_{T}-0.09{\mathrm{FP}}_{T}-0.35{\mathrm{WQ}}_{T}$$

(8) $${\mathrm{SW}}_{Y}=218.9+0.33{\mathrm{SE}}_{Y}-0.6{\mathrm{AT}}_{Y}-0.13{\mathrm{WQ}}_{Y}-0.01{\mathrm{WT}}_{Y}$$

(9) $${\mathrm{SE}}_{Y}=16.2+0.26{\mathrm{SW}}_{Y}+0.47{\mathrm{RE}}_{Y}+0.04{\mathrm{EP}}_{Y}$$

(10) $${\mathrm{SF}}_{Y}=13.579-0.07{\mathrm{SW}}_{Y}-0.38{\mathrm{SE}}_{Y}+0.53{\mathrm{FS}}_{Y}+0.78{\mathrm{FP}}_{Y}+0.72{\mathrm{WQ}}_{Y}.$$

For both the areas of Taoyuan and Yunlin, the endogenous variables of SW and SE have an impact on SF, while SE and SW have an influence on SW and SE only, respectively. As for the other independent variables, FS (farmer salary), AT (access to tap water), and RE (share of green energy) are the most significant variables for SF, SW, and SE in the Taoyuan area, respectively. In the Yunlin area FP (food price) and WQ (water quality) have a significant influence on SF. AT and RE have apparent impact on SW and SE, respectively.

In the analysis, the coefficients were determined empirically, which means that the obtained values delineate the correlation among the variables numerically without considering their physical meanings. For the sustainable water, energy, and food indices in the present study, the concept of sustainability is based on the comparison with another relative reference. For two different areas or scenarios, their sustainable indices were calculated on the same basis. Further judgement should be made for a scenario to be “more sustainable” or not in reference to the other scenario. Inspecting the obtained index individually does not result in an appropriate representation of its sustainability. Therefore, it is not appropriate to compare the numbers of SW, SE, SF, and SI resulting from two different calculations numerically, unless they were obtained under the similar premises. In this study, the sustainable indices were calculated and compared to demonstrate the relative sustainability of the two investigated areas.

The SI model was applied to calculating and comparing the sustainability of the two investigated areas with the consideration of population density, and the results are also shown in Table 4. In 2002, the Yunlin area is considered to be more sustainable than the Taoyuan area, but the difference in between is not as apparent as that in 2015. From 2002 to 2015, the SI for Yunlin exhibited a decreasing trend (i.e., moving towards a more sustainable direction) in general and that for Taoyuan showed an increasing trend (moving in a less sustainable direction). Because the Taoyuan area is a densely populated district, and resource consumption is more apparent and food production is less prevalent, because most of the land is used for industrial and commercial activities instead of agricultural purposes.

As reported in many studies, sustainability is a conceptual idea, which encourages the balancing utilization of environmental resources. In this study, an empirical framework for relative sustainability was proposed. Three indices for water, energy, and food were calculated individually for two different selected areas to compare their sustainability achievements. Furthermore, an integrated sustainable WEF index (SI), which considered food production, energy consumption, and water consumption, was proposed. According to the proposed formula, a higher value of the SI implied greater energy and water consumption and less food production, indicating a less sustainable situation compared to a scenario with a lower SI value. By inspecting the calculated sustainable index for different scenarios or areas, it can be concluded that the implemented policies or projects that result in higher SI values will make WEF resource utilization move in a more sustainable direction. This study also supports the fact the proposed framework is applicable to compare the sustainability achievements for two different projects or two different areas.

### 4.2. Scenarios Assessment

The calculated SIs for the three future scenarios (i.e., current practice enforcement, sustainable practice, and unsustainable practice) are shown in Table 5. In 2016, the SI in Taoyuan and Yunlin are 940.52 and 782.34. If the resource-consuming behaviors remain the same, the SIs would decrease 3.0% and 3.5% in Taoyuan and Yunlin in 2050, respectively. With the efforts to achieve the sustainable goals in 2050, the SIs decrease by 5.4% and 13.8% in Taoyuan and Yunlin in 2050, respectively. If an unsustainable development occurs in 2050, the SIs will increase by 30.7% and 15.2% in Taoyuan and Yunlin, respectively, as shown in Table 5.

According to the simulation results, the degree of sustainability of the two investigated areas can be enhanced in 2050 by implementing the following conditions: agricultural productivity and incomes of food providers are doubled and food prices should remain stable. In the sustainable water regression model, accessible and affordable water, increasing the ratio of treated wastewater, and reducing water pollution were important. In the sustainable energy regression model, affordable and renewable energy should be sufficiently supplied and the cost of the energy supply should maintain stable.

Based on the simulation results, three phenomena were observed. Firstly, the rural area has a lower SI (i.e., is more sustainable) than the urban area, whereas a more crowded area has a higher consumption of resources. Secondly, by comparing the SW estimation and equation coefficients, both areas are more sensitive to water resources. Lastly, food sustainability is less important in the urban areas and has little impact on the SI. The overall SI trend in the urban area smoothly increased (i.e., became less sustainable), while that of the rural area slightly decreased (i.e., became more sustainable) in the last decade (as shown in Table 4). The moving trends do not vary significantly and may be due to the improvement in the operation of water, energy, and food management, while the water supply, which is the primary resource almost for all the industries, fluctuated. From the simulation, the rural area showed a more sustainable trend for the utilization and management of WEF resources. However, the SI in the urban area slightly increased, and the affected population was much greater than that in the rural area.

### 4.3. Discussion

In the literature, various methodologies for nexus research have been reported. However, research assumption, goals, scales, and data availability are important to determine which approach should be used for the investigation of the water–energy–food nexus. Moreover, no single approach is applicable to all situations [39,51]. Among the reported studies, six key approaches (i.e., (1) life-cycle analysis, (2) system dynamics model, (3) investigation and statistical methods, (4) computable general equilibrium model, (5) econometric analysis, and (6) ecological network analysis) are used oftentimes to explore the complex correlation in the WEF nexus.

Life-cycle analysis (LCA) is a popular approach to quantitatively investigate the environmental impacts of a given product or process throughout its life cycle. It can accurately show the quantification of any unit during its life cycle and easily export its calculation processes, with the characteristics of identifying all of the inputs or outputs that may have significant impacts on environment [52]. LCA provides a reliable analytical framework and environmental data support for decision-making, which has been extensively applied to evaluate the environmental impact of nexus sectors across their production and consumption processes, seeking an effective way to deal with current resource shortages and climate change. System dynamics modeling (SDM) is a top-down modeling method, which assumes that system behaviors are determined by their structures, allowing for comprehensive analysis of multi-sectoral systems at both the macro and micro levels by establishing feedback loops among the elements within a given system. This character enables its adaptability for multidisciplinary problems [53]. Investigations and mathematical statistics, illustrating nexus issues through field surveys, expert discussion, and collections of public data released by local agencies and governments, and related literatures, are widely used to investigate the correlations between nexus sectors. Tracing resource consumption related to the production process of a certain resource could quantify assessments for the interactions between water, energy, food, and other resources. Adopting such methods, Machell et al. (2014) identified the electric power generation accounting for approximately 15% of the total global freshwater water withdrawals and around 70% of the global water consumption by food production [54]. The computable general equilibrium model (CGE) is an economic model that is frequently applied to policy analyses pertaining to the economy. The CGE models can explore the influences of policies on the nexus systems through price mechanisms by grasping the linkages pertinent to market behavior and changes [55,56]. Econometric analysis uses statistical methods to assess empirical content to economic relations by non-experimental economic data analysis [57]. This method of multiple regression analysis was deemed to be the foundation for econometric analysis. Ecological network analysis (ENA) was developed from input–output analysis and is one of the main methods for evaluating the interactions between economic and natural components. By integrating multiple entities connected by metabolic flows, ENA analyzes both direct and indirect flows in interwoven chains of production and consumption, showing the potential to investigate the trade-off between multiple elements [58,59].

WEF nexus evaluation framework is very important and complicated. The main challenge is to integrate the collected data and analyze the correlations of the involved sectors. Conversely, this study aimed to evaluate sustainability achievements under the implications of the WEF nexus for several given scenarios comparatively and empirically. An integrated evaluation of the WEF nexus was conducted for two areas of different levels of urbanization using empirical multiple linear regression in a simultaneous equation model. However, the application of the modeling framework requires further attention that the numbers obtained in one study should not be compared with those in another directly. A comparison can be made only when the calculations were conducted on the basis of data collected from the same period of time and utilized for regression analysis.

## 5. Conclusions and Policy Suggestions

Many WEF-related studies have been reported in the literature, and qualitative discussion has been made extensively to explore the nexus among WEF resources. However, little has been reported regarding investigating such a complicated nexus using simultaneous equation systems. Therefore, this study can be regarded as a pioneer study to quantify sustainability achievement by comparatively using simultaneous equation systems and multiple regression. The study demonstrated a feasible framework of comparative assessment of WEF sustainability. It is definitely not appropriate to compare these numbers if they are not obtained on the same fundamental assumptions and basis. Therefore, to evaluate a project or action plan, the evaluation using the proposed framework has to be conducted by constructing models for the given situations, and judgements must be made accordingly.

Exploring the WEF nexus enables the understanding of the complex relationship among WEF resources and the filling of the gap between strategy planning and policy implementation. This study constructed a sustainable WEF model using a simultaneous equation system to quantify the complex relationships in the WEF nexus, and each equation considered the impacts of the other two resources. Because resources are indispensable for human survival, population density is another key factor for sustainable WEF index calculation. In the sustainable water, energy, and food regression models, the coefficients were calculated to explain the weights of the variables. For the Yunlin area, water has the most significant impact on agricultural activities. The sustainable-WEF index was found to be capable of describing the sustainability of the urban and rural areas.

Providing safe, nutritious, sufficient, and affordable food is important. Because farming is affected by the degree of pollution of the soil, groundwater, and the environment, increasing the investment in agriculture technology to maintain the proper functionality of the ecosystem should be encouraged. Productivity should be enhanced to meet the needs of the increasing population. Water-saving crops should be promoted in cultivation to ensure stable agricultural production under the impact of climate change. The more intense precipitation and short rainy season in Taiwan caused the shortage of available surface water. Increasing the water-use efficiency in agricultural, industrial, and domestic sectors is necessary while the global population continues to grow. Ensuring the quality of water resources and water-related ecosystems must be enforced. Human activities have numerous impacts on the water environment, and wastewater should be recycled and reduced to improve the ambient water quality. Reliable and affordable energy is the foundation to maintain economic development. Power supply facilities that provide a variety of energy-related services have been the greatest pollution sources to the environment. Thus, the development of clean energy technology and the improvement of energy efficiency deserve immediate attention.

## Figures and Tables

**Table 1:** Descriptive statistics of each variable in the rural model (Yunlin area).

Variable	Definition	Unit	Average	Standard Deviation	Min	Max	Observations
SW_Y_	Water demand	10^7^ cubic meters	20.96	1.49	16.87	22.38	15
SE_Y_	Electricity demand	Million kWh	19.16	0.46	18.83	20.36	15
SF_Y_	Cultivated production	10^4^ ton	23.67	2.20	18.46	26.81	15
AT_Y_	Access to tap water	%	93.90	0.39	93.40	94.96	15
WQ_Y_	Water quality	RPI ^1^	3.59	0.53	3.10	5.10	15
WT_Y_	Wastewater treatment	%	23.95	8.19	10.41	35.22	15
RE_Y_	Share of green energy	%	3.53	0.38	3.00	4.30	15
EP_Y_	Energy price	NTD ^2^/KWh	2.46	0.35	2.05	3.07	15
FS_Y_	Farmer salary	10^5^ NTD/year	14.16	0.90	12.75	15.58	15
FP_Y_	Food price	NTD/kg	59.90	5.48	48.33	67.37	15

^1^ River Pollution Index; ^2^ New Taiwan Dollar.

**Table 2:** Descriptive statistics of each variable in the urban model (Taoyuan area).

Variable	Definition	Unit	Average	Standard Deviation	Min	Max	Observations
SW_T_	Water demand	10^7^ cubic meters	25.81	5.70	5.82	31.22	15
SE_T_	Electricity demand	billion kWh	26.30	1.67	22.67	28.44	15
SF_T_	Cultivated production	10^4^ ton	5.40	1.35	2.93	8.82	15
AT_T_	Access to tap water	%	94.52	0.91	92.69	95.44	15
WQ_T_	Water quality	RPI ^1^	5.02	0.79	3.40	6.30	15
WT_T_	Wastewater treatment	%	48.32	11.72	34.50	72.11	15
RE_T_	Share of green energy	%	3.53	0.38	3.00	4.30	15
EP_T_	Energy price	NTD ^2^/KWh	2.46	0.36	2.05	3.07	15
FS_T_	Farmer salary	10^5^ NTD/year	14.16	0.90	12.75	15.58	15
FP_T_	Food price	NTD/Kg	58.47	5.46	47.68	66.25	15

^1^ River Pollution Index; ^2^ New Taiwan Dollar.

**Table 3:** Simultaneous equations model regression results in Taoyuan and Yunlin.

Area	Taoyuan			Yunlin		
Variable	SW	SE	SF	SW	SE	SF
SF						
SW		−0.13	−0.25		0.26	−0.07
SE	−0.28		−0.73	0.33		−0.38
FS			0.21			0.53
FP			−0.09			0.78
AT	0.66			−0.6		
WQ	−0.27		−0.35	−0.13		0.72
WT	−0.54			−0.01		
RE		0.69			0.47	
EP_T_		0.1			0.04	
Intercept	43.65	20.95	22.43	218.9	16.27	13.579
Observation	15	15	15	15	15	15
*R*-square	0.4	0.7	0.2	0.4	0.4	0.6

**Table 4:** Results of the sustainable WEF index.

Year	Taoyuan				Yunlin				Sustainable WEF Index	
	PD_T_	SW_T_	SE_T_	SF_T_	PD_Y_	SW_Y_	SE_Y_	SF_Y_	Taoyuan	Yunlin
2002	1468.20	96.02	19.31	−6.13	575.44	167.46	22.17	58.28	885.66	834.75
2003	1492.34	95.27	19.73	−5.89	573.66	168.10	23.20	53.20	883.48	877.22
2004	1517.69	95.39	19.55	−7.18	570.77	168.00	23.34	56.19	894.52	857.80
2005	1540.04	95.83	19.98	−7.00	568.11	168.23	23.39	58.92	901.37	841.63
2006	1565.30	96.09	20.07	−7.60	564.36	168.42	23.53	58.12	910.36	847.91
2007	1584.80	96.39	20.17	−7.54	562.17	168.50	23.59	57.14	914.40	854.48
2008	1604.23	97.10	19.99	−6.53	560.63	168.36	23.61	62.79	912.36	817.59
2009	1620.69	96.25	20.21	−6.71	559.94	168.35	23.58	64.18	910.30	808.38
2010	1639.75	95.90	19.87	−8.05	555.96	168.27	23.52	62.69	916.55	816.00
2011	1648.96	95.76	19.99	−8.31	552.79	168.23	22.58	62.98	919.02	807.24
2012	1662.77	96.23	20.50	−8.08	550.80	168.09	24.06	64.74	925.62	804.13
2013	1674.12	95.76	20.91	−8.19	548.32	167.96	23.62	65.23	926.84	796.87
2014	1685.84	95.60	22.92	−4.04	546.43	168.19	23.45	66.84	910.62	786.67
2015	1724.70	95.89	20.18	−9.00	542.00	168.21	23.60	65.58	932.12	794.65
2016	1759.09	96.35	20.80	−8.72	538.31	168.27	23.88	67.74	940.57	782.34
Average	1612.57	95.99	20.28	−7.26	557.98	168.18	23.41	61.64	912.25	821.84

**Table 5:** Scenario simulation results.

Trends	SI Taoyuan	SI Yunlin
2016	940.52	782.34
Maintaining in 2050	912.43	754.58
Sustainable in 2050	725.47	614.35
Unsustainable in 2050	1229.25	901.45
